# Immune Response to *Enterococcus gallinarum* in Lupus Patients Is Associated With a Subset of Lupus-Associated Autoantibodies

**DOI:** 10.3389/fimmu.2021.635072

**Published:** 2021-05-28

**Authors:** Harini Bagavant, Antonina M. Araszkiewicz, Jessica K. Ingram, Katarzyna Cizio, Joan T. Merrill, Cristina Arriens, Joel M. Guthridge, Judith A. James, Umesh S. Deshmukh

**Affiliations:** ^1^ Arthritis and Clinical Immunology, Oklahoma Medical Research Foundation, Oklahoma City, OK, United States; ^2^ Departments of Medicine and Pathology, University of Oklahoma Health Sciences Center, Oklahoma City, OK, United States

**Keywords:** lupus, gut microbes, ribosomal P, microbiome, bacterial antibodies, autoantibodies, anti-RNA

## Abstract

Interactions between gut microbes and the immune system influence autoimmune disorders like systemic lupus erythematosus (SLE). Recently, *Enterococcus gallinarum*, a gram-positive commensal gut bacterium, was implicated as a candidate pathobiont in SLE. The present study was undertaken to evaluate the influence of *E. gallinarum* exposure on clinical parameters of SLE. Since circulating IgG antibodies to whole bacteria have been established as a surrogate marker for bacterial exposure, anti-*E. gallinarum* IgG antibodies were measured in banked serum samples from SLE patients and healthy controls in the Oklahoma Cohort for Rheumatic Diseases. The associations between anti-*E. gallinarum* antibody titers and clinical indicators of lupus were studied. Antibodies to human RNA were studied in a subset of patients. Our results show that sera from both patients and healthy controls had IgG and IgA antibodies reactive with *E. gallinarum*. The antibody titers between the two groups were not different. However, SLE patients with Ribosomal P autoantibodies had higher anti-*E. gallinarum* IgG titers compared to healthy controls. In addition to anti-Ribosomal P, higher anti-*E. gallinarum* titers were also significantly associated with the presence of anti-dsDNA and anti-Sm autoantibodies. In the subset of patients with anti-Ribosomal P and anti-dsDNA, the anti-*E. gallinarum* titers correlated significantly with antibodies to human RNA. Our data show that both healthy individuals and SLE patients were sero-reactive to *E. gallinarum.* In SLE patients, the immune response to *E. gallinarum* was associated with antibody response to a specific subset of lupus autoantigens. These findings provide additional evidence that *E. gallinarum* may be a pathobiont for SLE in susceptible individuals.

## Introduction

Regulated interactions between the immune system and microbes at mucosal surfaces play a critical role in maintaining immune homeostasis (1, 2). Under dysbiotic conditions, disruption of these interactions can manifest as a loss of immune tolerance and the development of autoimmunity. In SLE, patients show gut microbial changes with reduced microbial diversity and alterations in fecal and serum metabolites (3). Analyses from different patient cohorts show changes in *Firmicutes*/*Baciteroides* ratios, increase in *Lactobaccillaceae*, and expansion of specific bacteria like *Ruminococcus gnavus* in the gut (4–8). In some studies, antibodies to these gut bacteria are associated with increased autoantibody titers and lupus disease activity. Further, inflammatory processes influence the local gut micro-environment and have the potential to modulate the microbial composition on the mucosal surface (9). Thus, a continual interaction between local and systemic autoimmunity, gut mucosa, and microbiota may regulate disease evolution.

In addition to the gut, the bacterial community in the oral environment can also influence SLE. Indeed, bacterial species of oral microbiota origin are observed in the gut of SLE patients (10) Commensal oral bacteria like *Capnocytophaga* have the potential of stimulating lupus-antigen reactive T cells and autoantibodies through molecular mimicry (11, 12). In SLE patients, antibodies to specific periodontal pathogens like *A. actinomycetemcomitans* and *P. gingivalis* are associated with higher disease activity (13). IgG antibody titers against a bacterial strain indicate prior or ongoing exposure to that strain (14, 15). Thus, it is plausible that dysbiosis at different mucosal surfaces and the exposure of the immune system to specific commensal and/or pathogenic bacteria contribute to inflammatory responses and exacerbation of SLE.

The influence of gut bacteria on SLE pathogenesis have been successfully investigated in mouse models and specific bacterial strains that may be relevant in human disease have been identified (6, 7, 16). However, extrapolating the findings from inbred mouse strains to a highly diverse human population, in a heterogenous disease like SLE, remains a significant challenge. Recently, *Enterococcus gallinarum*, a gram-positive commensal bacteria present in the gut of lupus-prone (NZW x BXSB) F1 mice, has emerged as a candidate pathobiont for triggering SLE (16). Mono-colonization of the gut mucosa with *E. gallinarum* modulated adhesion molecules on the mucosal epithelium and allowed the migration of bacteria through the mucosa into the liver and systemic circulation in mice. *E. gallinarum* was also isolated from fecal samples and liver biopsies from patients with autoimmune hepatic disease and lupus patients with hepatic involvement. These patients showed a strong correlation between circulating antibodies to the bacterial RNA and human RNA, suggesting a causal relationship between the hepatic entry of *E. gallinarum* and SLE. However, this exciting observation was done in a limited number of SLE patients. In addition, whether *E. gallinarum* influences the clinical features of SLE in patients was unclear. To address these issues, we measured the levels of IgG and IgA antibodies to *E. gallinarum* (anti-*Eg) *in banked serum samples from a diverse and well-characterized cohort of SLE patients. Antibody responses to *E. gallinarum* were used as a surrogate marker of exposure to this bacteria,* *and the association between anti-*Eg* titers and clinical indicators of SLE was studied.

## Materials and Methods

### Study Design

The research was performed in accordance with the Helsinki Declaration and approved by the Oklahoma Medical Research Foundation Institutional Review Board. Banked serum samples and clinical data from SLE patients seen between May 2002 and October 2014 were obtained from the Oklahoma Rheumatic Disease Research Core Center (ORDRCC). The patients who met ≥4 of the 1997 modified American College of Rheumatology Classification Criteria for SLE (17, 18) were evaluated for disease activity and serum autoantibody profiles. The demographics of the patients (n=303) in this study are shown in **Supplementary Table 1**. Serum autoantibodies were measured using multiplex fluorescent bead-based assays. The antigens studied were dsDNA, chromatin, Ro/SSA, La/SSB, Sm, smRNP, RNP, RNP-A, RNP-68, Centromere B, Scl-70, and Ribosomal P. The antibody levels were quantified based on the fluorescence intensity for each specificity. The positive cut-off for the anti-dsDNA was set at 10 IU/mL (range 0- >300) and for all other specificities was 1.2 IU/mL (range 0- >8) per manufacturer’s recommendations. Clinical assessments of SLE were performed using the hybrid SELENA- SLE Disease Activity Index (SLEDAI) (19) and the British Isles Lupus Assessment Group (BILAG-2004) Index (20). Serum samples from de-identified healthy volunteers (n=66) were studied for antibodies to *E. gallinarum*, *E. faecalis*, and human RNA.

### Detection of Antibody to *Enterococci*

*Enterococcus gallinarum* (ATCC#BAA-748) and *Enterococcus faecalis* (ATCC#19433, Type strain) were obtained from the American Type Culture Collection (ATCC, Manassas, VA). These strains are utilized extensively as control/reference strains, and their use will allow for comparisons with studies performed by other investigators in future. The bacteria were cultured in Brain Heart Infusion broth, harvested, washed extensively with PBS, and stored as pellets in single-use aliquots at -80°C. An ELISA-based assay was used to measure antibodies to formalin-fixed whole bacteria as previously described (21). All sera from SLE patients and healthy controls were tested at a 1:500 dilution for anti-bacterial IgG and 1:100 dilution for anti-*E. gallinarum* IgA antibody titers. Serial dilutions from a pooled serum sample were included in each assay as a calibrator. A standard curve was constructed, and the titers of anti-bacterial antibody* *were calculated for each sample and expressed as units/mL.

### Detection of Antibodies to RNA

Human RNA was purified from THP1 (ATCC#TIB-202), a human monocytic cell line, propagated in RPMI-1640 with 10% bovine calf serum. RNA was extracted from THP1 cells using the RNeasy Mini Kit (Qiagen, Germantown, MD). Genomic DNA contaminants in the human RNA were eliminated by RNase-free DNase1 digestion using manufacturer’s protocols (Qiagen, Germantown, MD), followed by purification using RNeasy Mini columns (Qiagen, Germantown, MD).

Synthetic double-stranded RNA (poly I:C) HMW was purchased from Invivogen (San Diego, CA).

IgG antibodies to RNA [human RNA, and poly (I:C)] were measured using an ELISA. RNA (5μg/mL) dissolved in PBS with 1 mM EDTA was coated on DNA-BIND ELISA plates (Corning, Glendale, AZ) overnight at 4°C. After blocking, the plates were incubated with serum samples (1:100 dilution) for 2 hours. Bound antibodies were detected with HRP-conjugated goat anti-human IgG (Southern Biotechnology, Birmingham, AL) and enzyme activity determined by tetramethylbenzidine substrate (Bio-Rad Laboratories, Hercules, CA). The reaction was stopped with 2.5N sulfuric acid, and the absorbance was read at 450nm.

### Statistical Analysis

Graph Pad Prism 9.0 software (GraphPad Software, San Diego, CA) was used for statistical analyses. Anti-bacterial antibody titers were log_10_ transformed. Normality tests were performed on each dataset, and non-parametric tests were used for non-Gaussian distributions. Antibody titers between two groups were compared using a t-test for normal distributions or Mann-Whitney test for non-Gaussian distributions. Antibody titers between multiple groups were compared using a one-way ANOVA test, and Sidak’s multiple comparisons post-test determined adjusted p values. For non-Gaussian distributions, antibody reactivity in multiple groups was compared by the Kruskal-Wallis test, followed by Dunn’s multiple comparison post-test. Correlations were determined by Pearson’s method for normal distributions and Spearman’s method for non-Gaussian distributions. Proportions were compared by the Chi-square test. A p-value of <0.05 was considered significant. Post-hoc power calculations were performed using https://epitools.ausvet.com.au/.

## Results

### Higher Titers of Anti-*Eg* IgG Are Associated With Ribosomal P, dsDNA, and Sm Autoantibodies in SLE Patients

**Figure 1:**
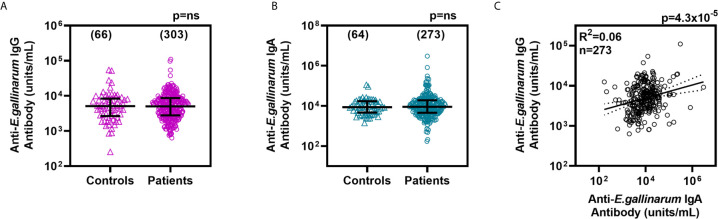
IgG anti-*Eg* **(A)** and IgA anti-*Eg* **(B)** titers in sera from healthy controls and lupus patients. Antibody titers are plotted as units/mL and the lines show median ± interquartile ranges. Correlation between IgG and IgA anti-*Eg* titers in lupus patients **(C)**. Each data point represents one serum sample and the number of samples studied are shown in parentheses. Antibody levels were compared by Mann-Whitney test and the correlation coefficient was determined by Pearson’s method. ns, not significant.

IgG antibody titers to formalin-fixed whole *E. gallinarum* bacteria were measured in sera from lupus patients (n=303) and healthy donors (n=66). Anti-*Eg* IgG were detected in all the sera tested, and the titers were not significantly different between the two groups (**Figure 1A**). The anti-*Eg* IgG titers between SLE patients based on self-reported race/ethnicities were also not different (**Supplementary Figure 1A**). Since *E. gallinarum* is associated with the gut mucosa, serum IgA antibody titers were also measured. No significant differences were seen in anti-*Eg* IgA titers between SLE patients and healthy donors or between patients in different racial/ethnic groups (**Figure 1B** and **Supplementary Figure 1B**). Anti-*Eg* IgG or anti-*Eg* IgA titers were not different between male and female patients (data not shown). No correlation was noted between age and anti-*Eg* IgG titers. However, anti-*Eg* IgA titers showed a statistically significant inverse correlation with age (Spearman r= -0.1941; p=0.0013). The anti-*Eg* IgG and IgA titers in the SLE patients showed a statistically significant, albeit modest, correlation (**Figure 1C**). The finding that anti-*Eg* IgG and IgA titers are not different suggests a comparable exposure to *E. gallinarum* in all groups.

**Figure 2:**
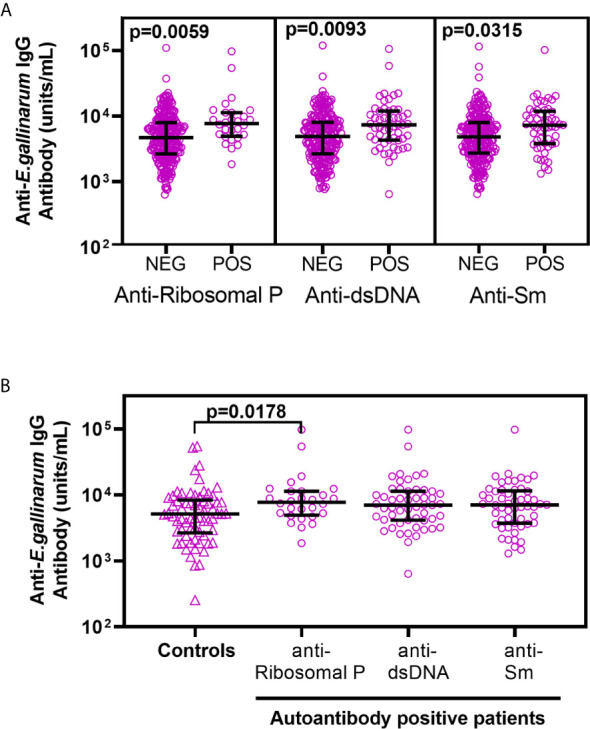
Lupus patients positive for anti-Ribosomal P, anti-dsDNA, and anti-Sm show significantly higher anti-*Eg* IgG titers **(A)**. SLE patients were stratified into autoantibody positive and autoantibody negative groups based on their reactivity to each antigen. The anti-*Eg* IgG titers were compared between the different groups using ANOVA, followed by Sidak’s multiple comparison’s post-test. The data from autoantibodies that failed to show significant association with anti-*Eg* IgG titers are shown in **Supplementary Figure 2**. A comparison of anti-*Eg* IgG titers in healthy controls with patients positive for Ribosomal P, anti-dsDNA, and anti-Sm using ANOVA followed by Sidak’s multiple comparison post-test **(B)**. Adjusted p values < 0.05 reaching statistical significance are shown.

Patients were stratified into groups based on the presence or absence of autoantibodies to different lupus-associated antigens. The anti-*Eg* titers between each autoantibody-positive and -negative group were compared (**Table 1**). As shown in **Figure 2**, higher anti-*Eg* IgG titers were associated with antibodies to Ribosomal P (p=0.0059), dsDNA (p=0.0093), and Sm (p=0.0315).

**Table 1:** Association between lupus autoantibodies and anti-bacterial IgG titers in SLE patients.

Autoantibody specificity			anti-*E. gallinarum* IgG					anti-*E. faecalis* IgG					anti-*S. gordonii* IgG				
			Median^@^	IQR*	p value^#^			Median	IQR	p value			Median	IQR		p value	
Ribosomal P	Neg	4732		5319	***0.0059***			6823	7462	0.1419			9311	8072		>0.9999	
	Pos	7745		6381				11066	15642				10666	4987			
dsDNA	Neg	4688		5097	***0.0093***			6546	7228	***0.0001***			9099	7276		0.1568	
	Pos	7015		7184				11776	16919				12823	8371			
Sm	Neg	4699		5066	***0.0315***			6310	6997	***0.0004***			9099	7589		0.6758	
	Pos	7047		7766				11749	14055				10789	6987			
chromatin	Neg	4688		5137	0.0694			6252	6974	***0.0021***			9226	6701		>0.9999	
	Pos	6124		6775				9954	13121				10447	7539			
SSA	Neg	4909		5336	0.7944			7129	7363	>0.9999			9727	7899		>0.9999	
	Pos	5929		5920				7870	10458				8750	9456			
SSB	Neg	5012		5537	0.8708			7396	8432	>0.9999			9705	8260		>0.9999	
	Pos	5861		7253				5702	8419				8318	6387			
SmRNP	Neg	4819		5368	0.0694			6026	6439	***<0.0001***			9099	6924		0.2978	
	Pos	5834		6642				10889	12498				10789	8304			
RNP	Neg	4909		5433	0.111			6310	7317	***0.0018***			9099	7477		0.4678	
	Pos	6209		6868				10889	13194				10789	8183			
RNP A	Neg	4909		5389	0.086			6397	7316	***0.0034***			9162	7571		0.7906	
	Pos	6368		7101				10889	13312				10568	7738			
RNP 68	Neg	4977		5828	0.7646			6653	7158	***0.0073***			9247	7606		>0.9999	
	Pos	6209		5262				12445	11464				12078	8895			
Centromere B	Neg	7047		7766	0.7947			7261	7678	>0.9999			9311	7917		>0.9999	
	Pos	6531		10186				7295	12884				11429	9627			
Scl 70	Neg	5000		5608	0.1727			7295	7983	>0.9999			9397	7866		>0.9999	
	Pos	13032		12903				7063	12541				14655	10246			
												

^@^Antibody Units/ml; *IQR, interquartile range; ^#^adjusted p value. Bold and underlined values indicate statistical significance (p<0.05).

A comparison between patients positive for anti-Ribosomal P, anti-dsDNA, or anti-Sm antibodies with healthy controls showed that anti-Ribosomal P reactivity in patients was consistently associated with higher anti-*Eg* IgG titers (adjusted p value = 0.0178; **Figure 2B**). Compared to healthy controls, higher anti-*Eg* IgG was also seen in patients with anti-dsDNA or anti-Sm following pair-wise analyses (**Supplementary Table 2**).

Statistical significance was not reached in comparisons of anti-*Eg* IgG titers between the other autoantibody-positive and -negative groups (**Supplementary Figure 2**) or between autoantibody positive patients and healthy controls (data not shown).

SLE patient categorization based on disease activity measures, including SLEDAI scores or BILAG indices, or clinical subsets failed to correlate with anti-*Eg* IgG titers. Similarly, the anti-*Eg* IgA titers failed to show association with the presence or absence of autoantibody specificity (**Supplementary Figure 3**), disease activity measures or clinical subsets (data not shown).

### Higher Antibody Titers to Gut Commensal Bacteria *E. Faecalis* and *S. Gordonii* Are Not Associated With the Presence of Anti-Ribosomal P Antibodies

To determine whether exposure to other Enterococci also shows associations with lupus autoantibodies, we measured IgG antibodies to *E. faecalis, *a commensal bacterium represented in the gut microbiome. Anti-*E. faecalis* IgG titers were not significantly different between healthy donors and SLE patients (**Supplementary Figure 4A**). Further, anti-*Eg*, and anti-*E. faecalis* IgG titers in patients showed a significant correlation (**Supplementary Figure 4B**), suggesting comparable exposure to the immune system and the possibility of cross-reactive antibodies.

Further analysis showed that anti-*E. faecalis* IgG titers were significantly higher in patients positive for antibodies to dsDNA, Sm, chromatin, and RNP autoantigens. However, the anti-*E. faecalis* titers between anti-Ribosomal P positive and negative patients failed to reach statistical significance (**Table 1**). A *post hoc* analysis showed that in this experiment, sample sizes gave >80% power to detect a significant difference in a two-tailed statistical test with a confidence level of 0.95. Thus, the negative result was likely not due to insufficient power, suggesting that the anti-Ribosomal P positivity and anti-*Eg* association is specific compared to *E. faecalis*-directed immune responses.

We have previously reported that in SLE patients, high titer antibodies to pathogenic periodontal but not commensal bacteria are associated with increased disease activity indices (13). *Streptococcus gordonii* is a gram-positive commensal bacterium present in dental plaque and also found in the gut mucosa. Anti-*S. gordonii* IgG titers failed to show significant associations with any of the lupus autoantibody specificities (**Table 1**).

### Reactivity to Ribosomal P and dsDNA Links Anti-Human RNA and Anti-*Eg* Antibodies in SLE Patients

**Figure 3:**
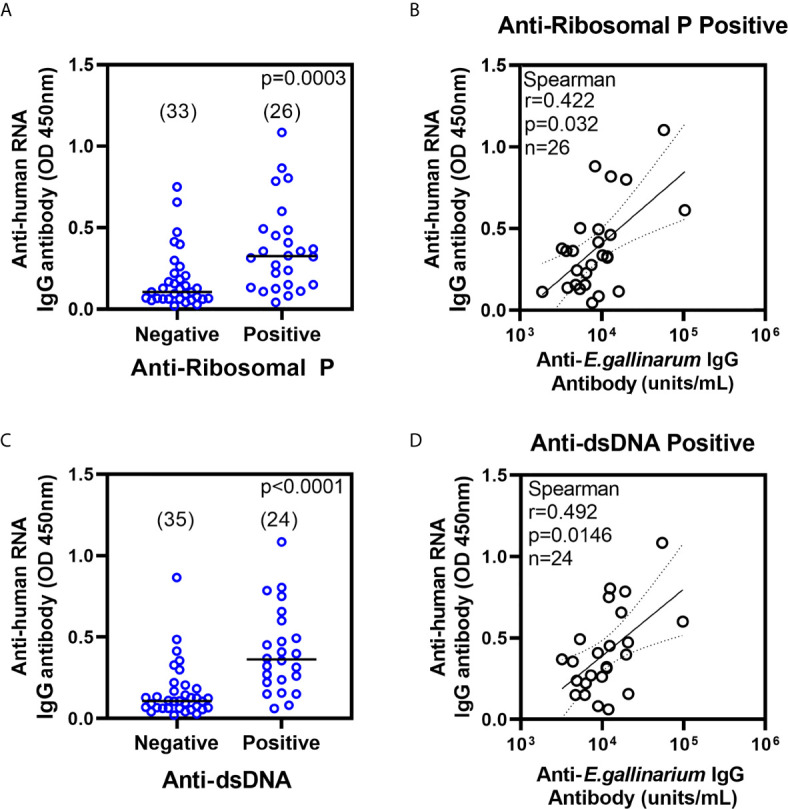
Anti-human RNA IgG antibodies in anti-Ribosomal P **(A)** and anti-dsDNA **(C)** negative and positive patients. All samples were tested at a 1:100 serum dilution and results are shown as absorbance at 450nm. Antibody levels were compared using non-parametric Mann-Whitney test. Number of samples are shown in parentheses. Correlation between anti-human RNA IgG and anti-*Eg* IgG titers in patients positive for anti-Ribosomal P **(B)** and anti-dsDNA **(D)**. OD, optical density.

A close association was reported between anti-Ribosomal P and anti-dsDNA in SLE patients (22, 23) and is replicated in our SLE patients (65% of anti-Ribosomal P positive patients are also anti-dsDNA positive). However, since ribosomes are closely bound to RNA, we postulated that the lack of immunoregulation in SLE patients would favor the presence of antibodies to human RNA in anti-Ribosomal P positive patients. To test this hypothesis, we purified RNA from a human monocytic cell line as a substrate to measure anti-human RNA in SLE patients who were Ribosomal P antibody positive (n=26) or randomly selected Ribosomal P negative (n=33). Patients positive for anti-Ribosomal P had higher anti-human RNA titers than anti-Ribosomal P negative patients (**Figure 3A**). Further, anti-human RNA titers in anti-Ribosomal P positive patients showed a modest but significant correlation with anti-*Eg* IgG antibody (Spearman r= 0.422, p=0.0319) (**Figure 3B**).

Anti-RNA antibodies in SLE patients also react with viral dsRNA and synthetic dsRNA (24). To investigate whether anti-RNA reactivity was skewed by RNA binding protein contaminants co-purified in the human RNA preparation, the same sera were screened for antibodies to synthetic dsRNA (poly I:C) coated on an ELISA plate. Anti-dsRNA reactivity was higher in Ribosomal P antibody-positive patients (**Supplementary Figure 5A**). The anti-human RNA and anti-dsRNA titers showed a strong correlation, Spearman r=0.782, p=2.57x10^-13^, n=59 (**Supplementary Figure 5B**), thereby confirming the reactivity to the nucleotide backbone.

Higher anti-RNA antibody titers are associated with higher disease activity (**Supplementary Table 3**) and a diversified autoantibody repertoire. Therefore, the association of anti-human RNA with anti-*Eg* titers might not be unique to Ribosomal P positivity. To investigate whether other autoantibody specificities also showed a similar relationship, patients were stratified into autoantibody-positive and -negative groups, and the correlation between anti-*Eg* and anti-human RNA titers in each group was studied (**Supplementary Table 4**). In addition to anti-Ribosomal P, anti-human RNA titers were also higher in patients positive for anti-dsDNA (**Figure 3C**). Further, anti-*Eg* IgG also showed modest but significant correlations with anti-human RNA titers in anti-dsDNA positive patients (r=0.492, p=0.0146). (**Figure 3D** and **Supplementary Table 4**).

## Discussion

The present study was prompted by a novel report describing the possible role of the pathobiont *E. gallinarum* in SLE pathogenesis (16). Since the Manfredo-Vieira et al. study was done in a limited number of lupus patients (n=15), we sought to investigate the role of *E. gallinarum* in a larger cohort of SLE patients (n=303). Furthermore, we also expanded the investigation into evaluating the association between *E. gallinarum* and multiple autoantibody specificities and SLE clinical parameters.

Using banked serum samples from a well-characterized cohort of SLE patients, our study demonstrates that IgG and IgA antibodies to *E. gallinarum* were present in lupus patients and healthy controls. Despite the differences in the numbers and characteristics of the patient populations, ELISA methodologies, and the specific bacterial strains, both studies showed comparable IgG and IgA anti-*Eg* titers between healthy controls and SLE patients. In our analysis, although anti-*Eg* titers did not correlate with either of the two disease activity indices (SLEDAI and BILAG), higher titers of anti-*Eg* IgG in patients were significantly associated with the presence of autoantibodies to Ribosomal P proteins, dsDNA, and Sm. In addition, only anti-*Eg*, but not anti-*E. faecalis* or anti-*S. gordonii* IgG antibody titers showed the strongest association with anti-Ribosomal P. Considered collectively, both studies suggest an involvement of *E. gallinarum*, and potentially other closely related enterococci, in SLE pathogenesis (3, 16). The analysis of gut microbiome in SLE patients from Guangzhou Province in China showed enrichment of the genus *Enterococcus* (3). Interestingly at species level, while this study reported an increase in *bacterium Te59R* (closely related *via* the 16S rRNA sequence to *Enterococcus faecium)*, it did not mention the detection of *E. gallinarum* in SLE patients. Whether lack of *E. gallinarum* reporting in this study is due to differences in patient demographics or/and methodology needs to be investigated in future.

Ribosomal P proteins are three highly conserved phosphorylated proteins on the 60s subunit of ribosomes and are a target for autoantibodies (25). Ribosomal P autoantibodies occur in a minority of lupus patients and in patients with autoimmune hepatitis (25, 26). In the present cohort, anti-Ribosomal P reactivity was seen in only 8.6% of the patients. Although anti-Ribosomal P antibodies are most frequently reported with neuropsychiatric lupus (27–29), they also identify a subgroup of patients at high risk of hepatic involvement. Studies by Stafford, Reichlin and colleagues showed that anti-Ribosomal P antibodies, if present, in healthy adults and children are masked and only detected following affinity purification on ribosome coated columns (30, 31). Thus, it is important to note that anti-Ribosomal P reactivity is highly specific for disease states, predominantly SLE, and is not detectable in sera from healthy individuals as reported in multiple studies (22, 32–35).

Ribosomal P protein is expressed on the cell membrane and can bind to sera from lupus patients (36). Ribosomal P antibodies can penetrate live hepatoma cells and block protein synthesis leading to cellular injury (37). Furthermore, we also noted higher anti-human RNA antibody titers in patients positive for anti-Ribosomal P. Considering that *E. gallinarum* was detected in liver biopsies from lupus patients and anti-*Eg* IgG was unique in its association with antibodies to Ribosomal P, it can be surmised that *E. gallinarum* mediated hepatic and/or systemic inflammation may contribute to anti-Ribosomal P autoimmune responses in some SLE patients. Whether this occurs through molecular mimicry or intermolecular epitope spreading will be tested in future studies by longitudinal analysis of serum samples from lupus patients and by developing experimental mouse model systems.

We have previously reported associations between the lupus autoantibodies and higher titers to the dental plaque bacteria *A. actinomycetemcomitans* and *P. gingivalis* implicated in periodontal disease (13). It is interesting to note that the antibodies to these oral pathogens were not different in patients with or without Ribosomal P reactivity. *A. actinomycetemcomitans* and *P. gingivalis *secrete virulence factors, invade the periodontal tissues, migrate to distant organs and cause inflammation (38, 39). In contrast, *E. gallinarum* is a commensal gut resident bacterium that can translocate to the liver. Taken together, these results suggest that the mechanism(s) of how periodontal and gut bacteria influence lupus might be different.

Some limitations of the present study include the unavailability of stool samples for microbiome analysis, a lack of patient medication history, and the absence of demographic data on the healthy controls. However, this study reinforces previous reports by our group and others (40, 41) that in retrospective studies of large and diverse patient cohorts, evaluating serum antibodies to pathogenic and commensal bacteria is a valuable tool to investigate the interaction between the microbial environment and autoimmunity. These data provide a rationale for performing metagenomic analyses of mucosal microbial communities in diverse SLE patient cohorts.

## Data Availability Statement

The original contributions presented in the study are included in the article/**Supplementary Material**. Further inquiries can be directed to the corresponding author.

## Ethics Statement

The studies involving human participants were reviewed and approved by Oklahoma Medical Research Foundation Institutional Review Board. The patients/participants provided their written informed consent to participate in this study.

## Author Contributions

HB designed and performed experiments, analysed the data, and wrote the manuscript. AA, JI, and KC performed experiments and analysed data. JM, CA, and JJ contributed to study design, acquisition of clinical data, data analysis, and writing of the manuscript. JG contributed to study design, critical review of data analysis, and writing of the manuscript. UD conceived of the idea, designed experiments, and wrote the manuscript. All authors contributed to the article and approved the submitted version.

## Funding

This work was supported by National Institute of Allergy and Infectious Disease (UM1AI144292), National Institute of Arthritis, Musculoskeletal and Skin Diseases (P30AR073750), and National Institute of General Medical Sciences (U54GM104938) of the National Institutes of Health; the Oklahoma Center for the Advancement of Science and Technology (HR15-145); and Institutional Funds from the Oklahoma Medical Research Foundation. JI was supported by the Jeff Metcalf fellowship grant. The content is solely the responsibility of the authors and does not necessarily represent the official views of the National Institutes of Health or the United States government.

## Conflict of Interest

The authors declare that the research was conducted in the absence of any commercial or financial relationships that could be construed as a potential conflict of interest.
