# Supplementary material for: Immune Response to Enterococcus gallinarum in Lupus Patients Is Associated With a Subset of Lupus-Associated Autoantibodies
Source: Front Immunol. 2021 May 28;12:635072. doi: 10.3389/fimmu.2021.635072 (PMC8193979; doi:10.3389/fimmu.2021.635072)
Supplement: Supplementary file 1 [file DataSheet_1.docx]

Supplementary Material

**Supplementary Table 1**

**Oklahoma Cohort for Rheumatic Diseases patient demographics**

|  | SLE patients |
| --- | --- |
| Total, n | 303 |
| Female, n (%) | 280 (92.4) |
| Age in years, median (range) | 39 (16-71) |
| Race, n (%) |  |
| Caucasian | 161 (53.1) |
| African American | 62 (20.4) |
| Asian | 13 (4.3) |
| American Indian | 40 (13.2) |
| Mixed (2 or more) | 26 (8.6) |
| Pacific Islander | 1 (0.3) |
| ACR SLE criteria, median (range) | 6 (4-11) |
| SLEDAI, median (range), n | 6 (0-24), 285 |
| BILAG total, median ( range), n | 10 (0-42), 284 |
| Clinical subsets, n (%) |  |
| Malar Rash | 188 (62) |
| Discoid Rash | 56 (18.5) |
| Photosensitivity | 201 (66.3) |
| Oral Ulcer | 217 (71.6) |
| Arthritis | 268 (88.4) |
| Serositis | 116 (38.3) |
| Renal | 59 (19.5) |
| Neurologic | 49 (16.2) |
| Hematologic | 183 (60.4) |
| Immunologic | 266 (87.8) |

**Supplementary Table 2**

**Anti-*Eg* IgG titers in sera from healthy controls and autoantibody positive lupus patients.**

|  | **Controls** | **Patients (autoantibody positive)** | | |
| --- | --- | --- | --- | --- |
|  |  | **Anti-Ribosomal P** | **Anti-dsDNA** | **Anti-Sm** |
| Median^@^ | 5117 | 7745 | 7015 | 7047 |
| IQR* | 5651 | 6381 | 7184 | 7766 |
| p value^#^ |  | ***0.0088*** | ***0.0286*** | ***0.0406*** |

^@^ anti-*Eg* IgG Antibody Units/ml; *IQR, interquartile range; ^#^ p value compared to controls.

**Supplementary Table 3.**

**Association between disease activity and anti-human RNA IgG reactivity**

| Disease activity index | Anti-human RNA negative | Anti-human RNA positive | p value |
| --- | --- | --- | --- |
|  |  |  |  |
| **SLEDAI** | n=29 | n=24 |  |
| 0-2 | 41.3% (12) | 4.2% (1) |  |
| 3-9 | 41.3% (12) | 54.2% (13) |  |
| >10 | 17.2% (5) | 41.6% (10) | ***0.0049*** |
|  |  |  |  |
| **BILAG** | n=29 | n=24 |  |
| Mild (C/D) | 44.8% (13) | 16.7% (4) |  |
| Moderate ( 1-2B) | 44.8% (13) | 50.0% (12) |  |
| Moderately severe  - severe (>3B or 1A) | 10.3% (3) | 33.3% (8) | ***0.0357*** |

Patients were stratified into anti-RNA IgG positive and negative groups using an ROC curve generated positive cut off value of 0.163 (91.7% specificity and 49.2% sensitivity). Disease activity was determined by SLEDAI and BILAG and data are shown as frequency % (number of patients) in each category. p values determined using Chi-Square test, p<0.05 was considered significant.

**Supplementary Table 4.**

**Correlation between anti-*Eg* IgG and anti-human RNA IgG antibody titers**

|  | **Autoantibody positive** | | | **Autoantibody negative** | | |
| --- | --- | --- | --- | --- | --- | --- |
| **Autoantibody**  **Specificity** | Spearman r | p | n | Spearman r | p | n |
| dsDNA | 0.492 | ***0.0146*** | 24 | -0.111 | 0.5246 | 35 |
| Ribosomal P | 0.422 | ***0.0319*** | 26 | 0.238 | 0.1828 | 33 |
| Chromatin | 0.287 | 0.0925 | 37 | 0.305 | 0.1682 | 22 |
| SSB | 0.321 | 0.2257 | 16 | -0.137 | 0.3807 | 43 |
| RNP A | 0.236 | 0.3033 | 21 | 0.113 | 0.4983 | 38 |
| SmRNP | 0.079 | 0.6673 | 32 | 0.183 | 0.3610 | 27 |
| SSA | 0.114 | 0.5621 | 28 | 0.060 | 0.7489 | 31 |
| RNP | 0.086 | 0.6755 | 26 | 0.082 | 0.6511 | 33 |
| Sm | 0.007 | 0.9722 | 26 | 0.153 | 0.3949 | 33 |

Patients were stratified into autoantibody positive and negative for each specificity. Spearman correlation between anti-*Eg* IgG and anti-human RNA IgG titers was determined for each group. p<0.05 was considered significant. n, number of patients.


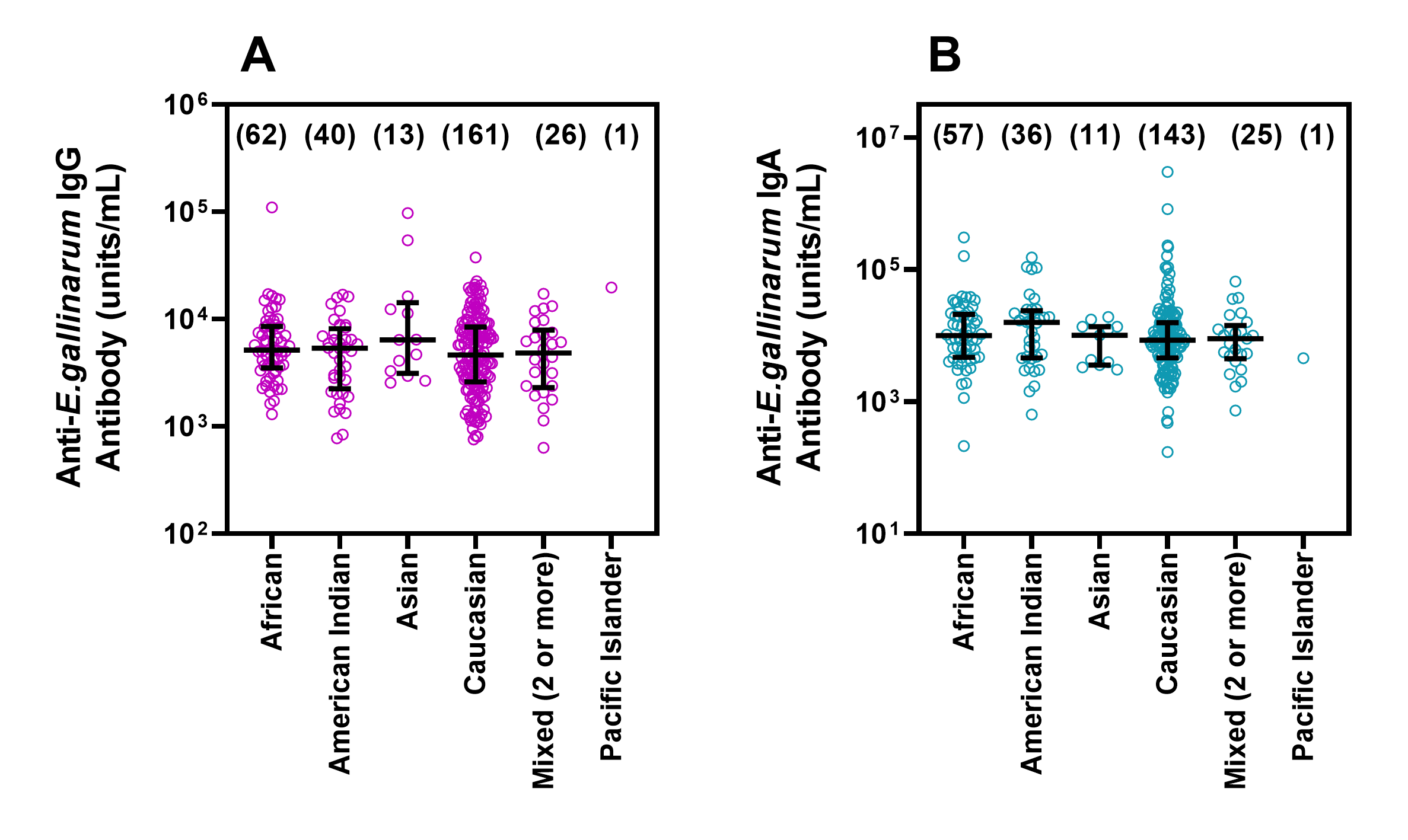


**Supplementary Figure 1:** Anti-*Eg* IgG **(**A**)** and anti-*Eg* IgA **(B)** titers in sera from lupus patients stratified by self-reported race/ethnicity. Antibody titers are plotted as units/mL and the lines show median + interquartile ranges. Each data-point represents one serum sample and the number of samples studied are shown in parenthesis.


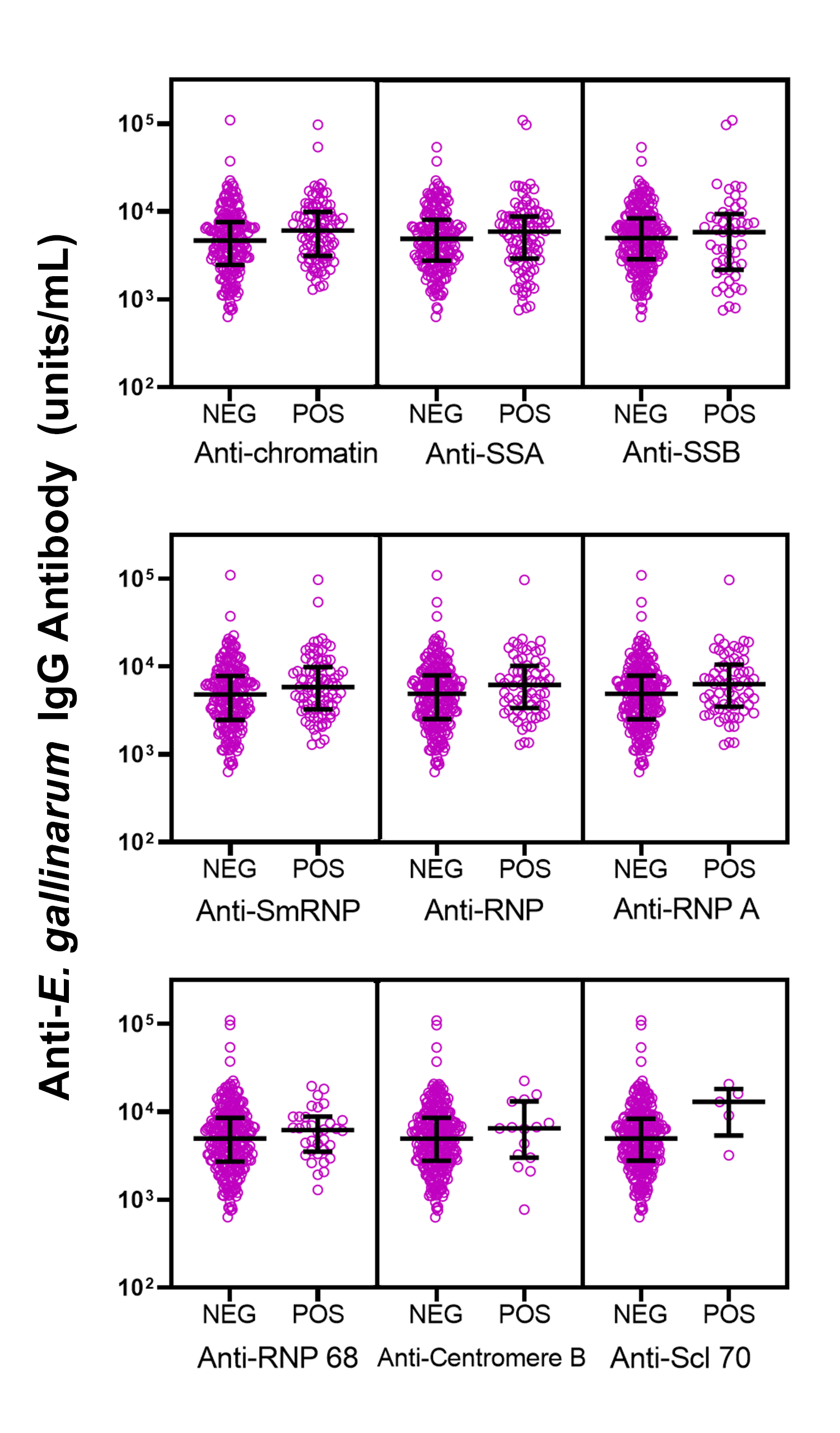


**Supplementary Figure 2:** Association between anti-*Eg* IgG titers with presence or absence of autoantibodies to the lupus-associated antigens. SLE patients were stratified into autoantibody positive and autoantibody negative groups based on their reactivity to each antigen. Autoantibody specificities that failed to show significant association with anti-*Eg* IgG titers are shown (adjusted p>0.05).


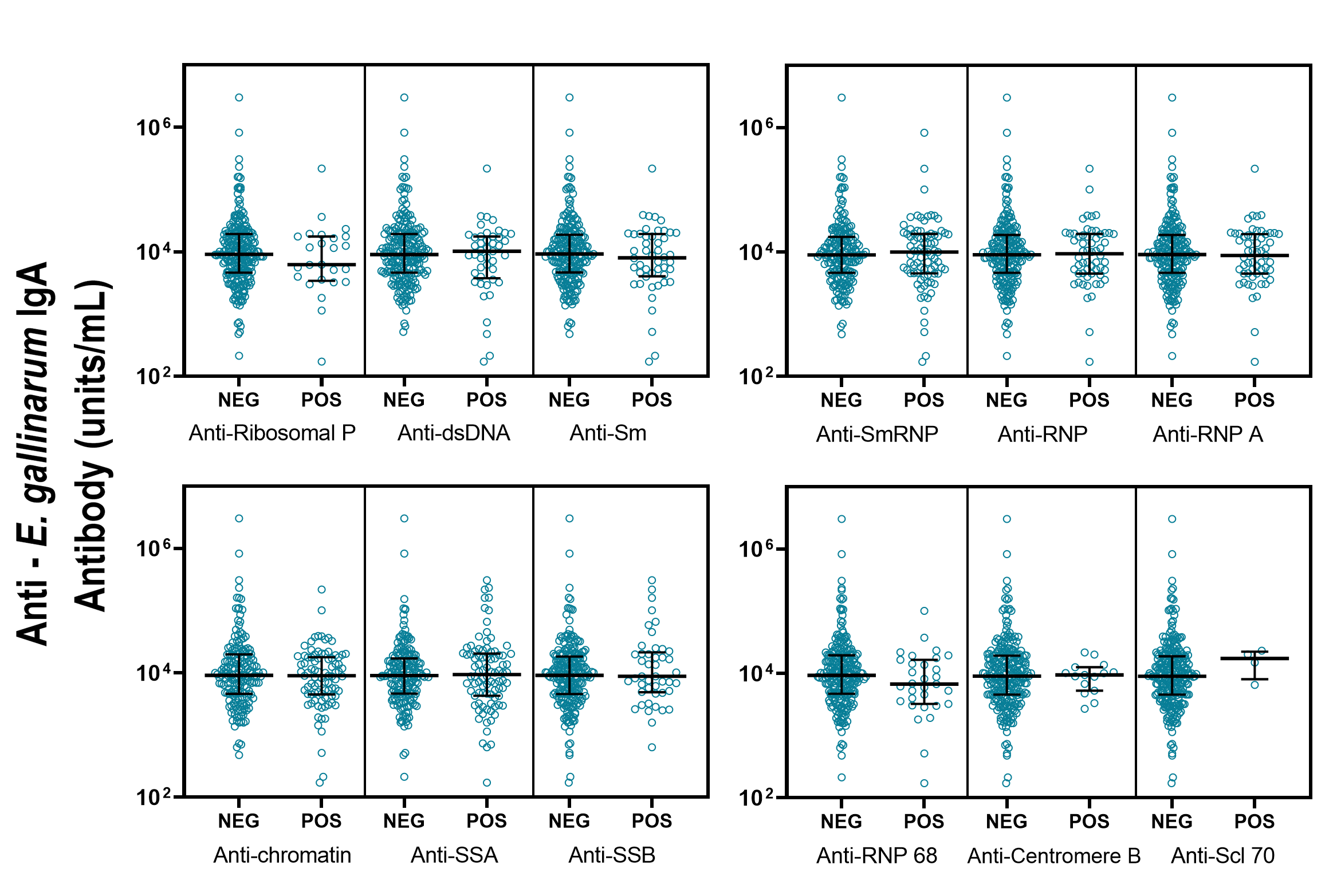


**Supplementary Figure 3:** Association between anti-*Eg* IgA titers with presence or absence of autoantibodies to the lupus associated antigens. SLE patients were stratified into autoantibody positive and autoantibody negative groups based on their reactivity to each antigen. All autoantibody specificities studied failed to show significant association with anti-*Eg* IgA titers (adjusted p>0.05).


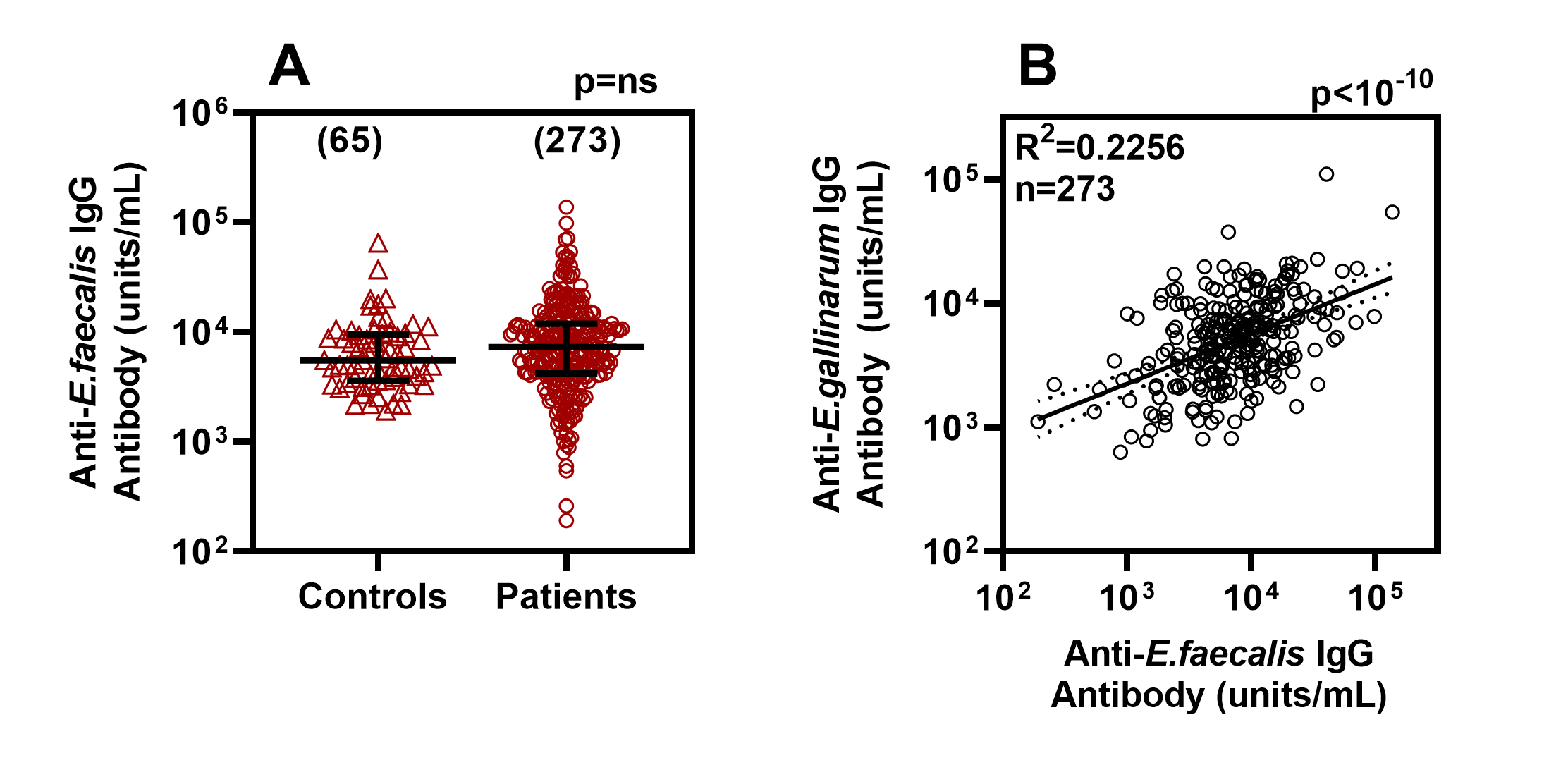


**Supplementary Figure 4:** Anti-*E. faecalis* IgG antibody titers in sera from lupus patients and healthy donors **(A)**. Antibody titers are plotted as units/mL and the lines show median + interquartile ranges. Each data-point represents one serum sample and the number of samples studied are shown in parenthesis. **(B)** Correlation between anti-*Eg* IgG and anti-*E. faecalis* IgG titers. Correlation coefficient calculated by Pearson’s method.


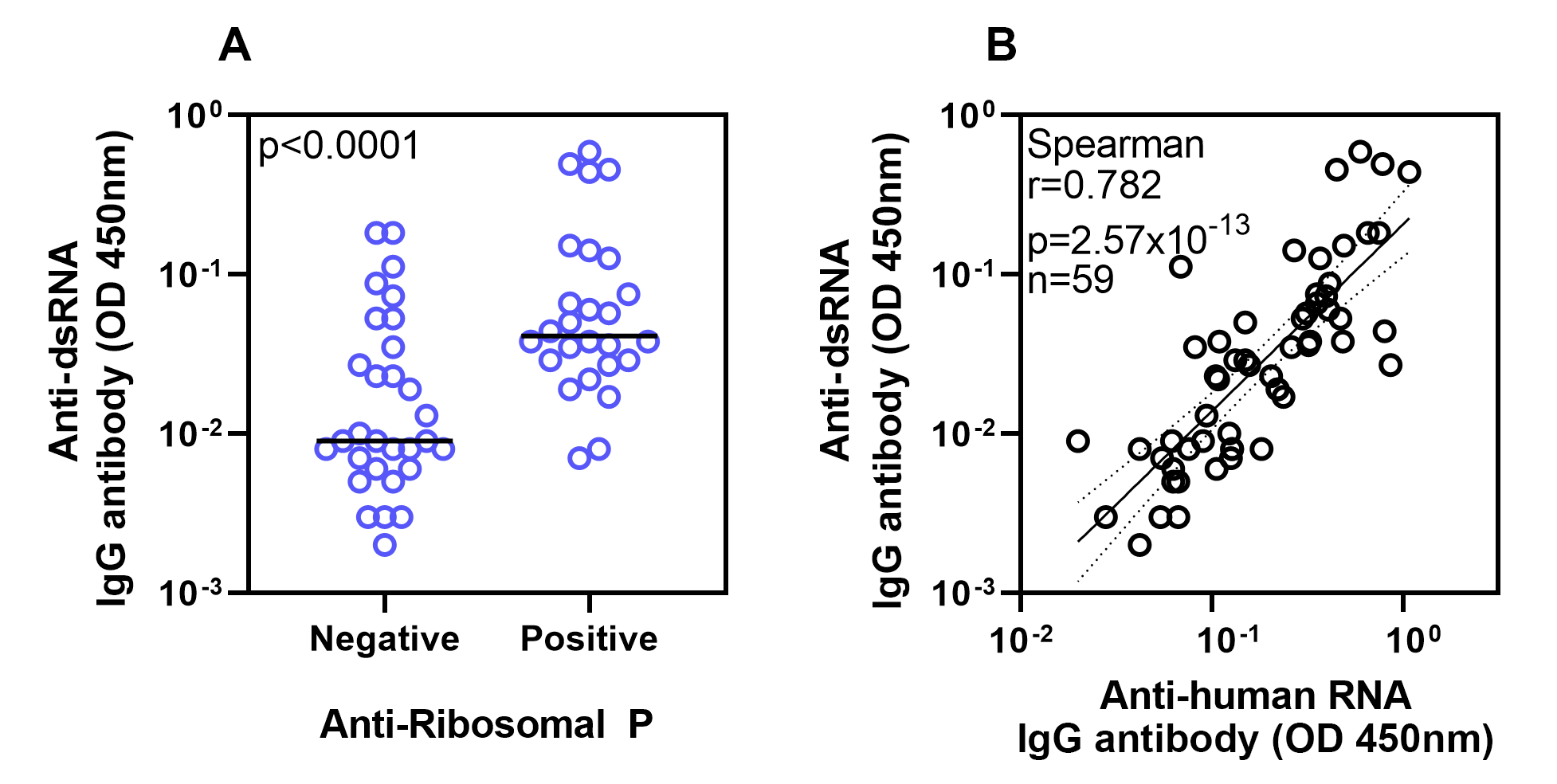


**Supplementary Figure 5.** Anti-dsRNA IgG antibodies in anti-Ribosomal P positive and negative patients **(A).** All samples were tested at a 1:100 serum dilution and results are shown as absorbance at 450nm. Antibody levels were compared using non-parametric Mann-Whitney test. Correlation between anti-human RNA IgG and anti-dsRNA IgG titers **(B)**. OD, optical density.
